# Ulnar Nerve Entrapment Among Cell Phone Users: Cell Phone Elbow (Cubital Tunnel Syndrome)

**DOI:** 10.7759/cureus.55500

**Published:** 2024-03-04

**Authors:** Kavitha Ukkirapandian, Sundaravadivel VP, Archana S Pawar, Karthika Priyadharshini Udaykumar, Muthulakshmi Rangasmy

**Affiliations:** 1 Physiology, Meenakshi Medical College Hospital and Research Institute, Meenakshi Academy of Higher Education and Research (MAHER) University, Chennai, IND; 2 Physiology, Meenakshi Medical College Hospital and Research Institute, Meenakshi Academy of Higher Education and Research (MAHER) University, Kanchipuram, IND; 3 Physiology, Sri Venkateswaraa Medical College Hospital and Research Institute, Chennai, IND; 4 Physiology, Jawaharlal Institute of Postgraduate Medical Education and Research, Pondicherry, IND

**Keywords:** wartenberg's sign, froment's sign, cubital tunnel syndrome, nerve conduction study, cell phone elbow, ulnar nerve

## Abstract

Introduction

Cell phone usage has tremendously increased, and to make usage comfortable, accessories such as Bluetooth earphones are available. But still, most people use cell phones for a long period of time by flexing their elbows near their ears. When the users flex the elbow to hold the phone near the ear, this results in increased pressure over the ulnar nerve since the ulnar nerve runs superficially at the level of the elbow. The extensive pressure over the ulnar nerve may result in nerve compression, which results in cubital tunnel syndrome, recently called the cell phone elbow. Hence, this study was undertaken to assess the ulnar nerve function among cell phone users in relation to the duration of usage.

Materials and methods

Young healthy volunteers (n = 30) aged between 20 and 25 years were selected for the study in order to prevent age-related neuropathic changes. After getting a history of mobile phone usage, the subjects were asked about neural symptoms such as tingling, numbness, and pain while using cell phones. Ulnar nerve function was assessed by Froment's sign and Wartenberg's sign. An ulnar nerve conduction study was done.

Results

Seventy percent of the subjects (n = 21) out of the 30 subjects participating in the study reported tingling and numbness during mobile phone usage. But Froment's sign and Wartenberg's sign were negative for all the subjects. There was a significant positive correlation (r = 0.913 and r = 0.8253) between the duration of mobile phone use and latency and a negative correlation (r = -0.8439) with conduction velocity.

Conclusion

The malposition of the elbow during prolonged cell phone use results in ulnar nerve entrapment. The continuous usage of cell phones without rest by flexing the elbow causes nerve compression. This can be taken as a warning sign to prevent further damage.

## Introduction

Mobile phone usage has increased exponentially, with broad usage ranging from oral communication to marketing, social media, mobile shopping, etc. To make usage comfortable while using the mobile phone, so many accessories, such as Bluetooth and earphones, are available. But even then, youngsters probably use their mobile phones to talk by flexing their elbows near the ear. Many research studies have been published that focus on the ill effects of cell phone usage on health, including radiation exposure, cancer [1,2], and mental health illnesses [3]. Though few studies have been conducted on the prolonged maintenance of the same posture and ulnar nerve function, this study was planned to correlate the duration of mobile phone use per day with ulnar nerve function.

In the upper extremities, after carpal tunnel syndrome, ulnar nerve entrapment is the second most prevalent compression neuropathy [4]. Cubital tunnel syndrome is the name given to the condition where the ulnar nerve is squeezed at the elbow behind the medial epicondyle of the humerus. A recent study conducted using fresh frozen cadaveric elbows demonstrated that the use of a cell phone-like activity is associated with excessive elbow and shoulder motions and results in an average strain of 6.3% [5]. Hence, this study was undertaken to assess the ulnar nerve function among cell phone users.

## Materials and methods

This is a cross-sectional observational study that was carried out at Sri Venkateswaraa Medical College and Research Centre after getting the Institutional Ethical Committee's approval (approval number: 052/2018-19). Healthy young subjects aged between 20 and 25 years and with a body mass index (BMI) of <25 kg/m^2^ were recruited for the study; their medical history and their cell phone usage history were collected. The habit of using headphones and Bluetooth headsets for mobile use and a history of upper limb injuries and peripheral neuropathies and systemic diseases such as diabetes, hypertension, and thyroid disorders were excluded from the study. Informed written consent was obtained from all the subjects. The total number of subjects who participated in this study was 30 males.

A set of questions were asked to know about cell phone usage, and the maximum duration of cell phone use per time without taking rest in a day was calculated for all the subjects. They were asked to report numbness, tingling, and paresthesia over the small finger, ring finger, and hypothenar eminence after using the phone for a long period of time.

As a part of the physical examination to rule out ulnar nerve entrapment, Froment's test and Wartenberg's sign were elicited.

Froment's test

To examine the adductor pollicis innervated by the ulnar nerve, the subjects were asked to hold a piece of paper between the thumb and radial side of the index finger. During this test, normally, the subject adducts the thumb by using the adductor pollicis, but if the subject flexes the thumb instead of adducting at the interphalangeal (IP) joint, it indicates a dysfunction of the ulnar nerve and constitutes a positive Froment's sign [6,7].

Wartenberg's sign

The subjects were asked to fully adduct their fingers at the metacarpophalangeal joint and proximal interphalangeal and distal interphalangeal joints in full extension. While doing this, the small finger drifts away from the others into abduction, which is considered Wartenberg's sign positive. When the palmar interossei muscle is affected, it results in a positive sign [6,8].

Ulnar nerve conduction

After the physical examination, a motor nerve conduction study for the ulnar nerve was done, and the subjects were fully oriented about the procedure and its use. "NeuroStim-NS2" (Medicaid Systems, Mohali, India) was used to record the ulnar nerve function. The subjects were seated comfortably in chairs, and the active electrode was placed over the hypothenar eminence, and the reference electrode was placed just 2 cm above the active electrode on the little finger. Electrical stimuli were given at the level of the wrist (stimulus 1 {S1}) and elbow (stimulus 2 {S2}). The distance between the stimuli was measured to calculate conduction velocity. Nerve conduction velocity is assessed by giving electrical stimuli at 10 mV, which is the minimum electrical current level at which the subjects feel stimuli as electrical stimuli and not as pain.

Statistical analysis

Descriptive statistics were used to tabulate the sensory changes with mobile phone usage. The Pearson regression analysis was done using the GraphPad Prism 8 version (GraphPad Software, San Diego, CA) to relate the ulnar nerve conduction with the duration of mobile phone usage per day.

## Results

Demographic data showed that all the subjects belonged to the young adult age group, and their BMI fell within <25 kg/m^2^, which indicates a healthy normal weight. The duration of mobile use is around 130 minutes, which indicates that the subjects have a history of continuous mobile use for more than three hours without taking a rest (Table 1).

**Table 1:** Demographic data and mobile usage BMI, body mass index; SD, standard deviation

Demographic data	Mean ± SD
Age (years)	21.2 ± 3.5
BMI (kg/m^2^)	23.6 ± 1.8
Duration of phone use (minutes) per day	130.4 ± 12

The frequency distribution table of sensory changes indicates that out of 30 subjects, 70% (n = 21) had complaints of paresthesia, and 20% (n = 6) had complaints of pain when they used the phone continuously for a longer duration of time. Ten percent (n = 3) of the subjects reported no sensory changes after using the cell phone (Table 2).

**Table 2:** Frequency distribution of sensory changes and mobile use

Sensory changes	Frequency distribution	Percentage (%)
Paresthesia (numbness and tingling sensation)	21	70%
Pain	6	20%
No sensory changes	3	10%
Froment's test: positive	0	0%
Wartenberg's sign: positive	0	0%

The latency time between the first stimuli and the response was around 4.8 ± 2.4 seconds, and the latency time between the second stimuli and the response was around 5.2 ± 1.7 seconds. The mean conduction velocity among mobile phone users is 56.2 ± 4.9 m/second (Table 3).

**Table 3:** Descriptive statistics of motor nerve conduction study S1, stimulus 1; S2, stimulus 2; SD, standard deviation

Motor nerve conduction study: ulnar nerve	Mean ± SD
Latency (S1) (seconds)	4.8 ± 2.4
Latency (S2) (seconds)	5.2 ± 1.7
Conduction velocity (m/second)	56.2 ± 4.9

The Pearson regression analysis demonstrated that the latency period for S1 and S2 stimuli was positively correlated with the duration of mobile phone usage. The conduction velocity of the ulnar nerve was negatively correlated with the duration of mobile phone use (Table 4).

**Table 4:** Regression analysis of the duration of mobile phone use and nerve conduction study r, Pearson regression analysis; S1, stimulus 1; S2, stimulus 2

Motor nerve conduction study: ulnar nerve	Duration of mobile phone usage	
	r	p
Latency (S1)	0.913	<0.0001
Latency (S2)	0.8253	<0.0001
Conduction velocity (m/second)	-0.8439	<0.0001

## Discussion

In this study, the nerve conduction report revealed that the duration of handling a mobile phone has a significant effect on ulnar nerve function, which was evidenced by prolonged latency and delayed conduction velocity in the ulnar nerve conduction study with an increase in the duration of cell phone use.

According to recent reports, electrodiagnostic studies, such as nerve conduction studies and electromyography (EMG), are crucial tools in the diagnosis [9-11] of ulnar neuropathy in addition to physical examinations. So, in this study, the early screening of ulnar nerve entrapment among cell phone users was planned. The mean motor conduction velocity recorded in this study among cell phone users was around 56 ± 4.9 m/second, which was lower than the normative data reported by Haghighat et al. (2018) [12]. Prolonged latency (>4 seconds for both S1 and S2) and the lowered conduction velocity of the ulnar nerve indicate nerve compression.

Three distinct locations in the elbow region may cause compression injuries to the ulnar nerve: the retro-epicondylar groove, the cubital tunnel, and the site where the nerve exits. In elbow flexion, the cubital tunnel progressively narrows and increases the pressure to >200 mm Hg [13]. The ulnar nerve is smooth and spacious during elbow extension, but it becomes flattened, tortuous, narrow, and inhospitable during elbow flexion [14]. With extreme flexion of the elbow, the medial head of the triceps pushes against the nerve posteriorly, additionally narrowing its passageway and fostering subluxation [15]. This anatomical relationship explains why the ulnar nerve is at risk of injury at the elbow [16].

Sardelli et al. mentioned that, among all contemporary tasks of daily living, cellular phone usage causes greater flexion at the elbow (flexion arc of 142°) than other tasks (flexion arc of 130°) [17]. Byl et al. documented that the ulnar nerve strain was 2% with 135° elbow flexion [18]. While using a cell phone for a prolonged period of time with elbow flexion, the ulnar nerve is placed in tension; the nerve itself can elongate from 4.5 to 8 mm. It narrows the space available for the nerve and increases the pressure within the cubital tunnel [9]. There is strong literature evidence that when the degree of elbow flexion increases, the pressure over the ulnar nerve also increases at the cubital tunnel [19-21], which supports these study findings.

The zero percentage of positive cases in Froment's test and Wartenberg's sign recorded in this study explain that ulnar nerve compression due to cell phone use has not resulted in nerve injury. This may be because all the subjects were young adults without comorbidities, and exposure to cell phones was not more than three years. Our findings were also supported by Vinitpairot et al. (2019), who stated that less strain activities can increase high strain in patients with a history of nerve scars and cubital tunnel syndrome [5]. Though there are studies to relate ulnar nerve compression with the degree of elbow flexion, to our knowledge, this is the first study to demonstrate the changes in ulnar nerve function in relation to the duration of cell phone use among youngsters.

Limitation

Along with the nerve conduction study, EMG could also have been done to ensure the muscle status. Since the needle electrode has to be used for EMG to avoid needle insertion-related discomfort, EMG was not done.

## Conclusions

This study will be useful to create awareness about the overuse of mobile phones among the young generation. We can recommend reducing mobile phone use, at least using a headset, or switching hands frequently instead of flexing the elbow. Though only nerve compression was documented, not nerve injury among cell phone users, this could be due to the restriction of the subjects to young age groups without any comorbidities. This study can be extended in the future to include people with comorbidities who are at risk of developing nerve compression.
